# Epigenetic networks coordinate DNA methylation across the genome

**DOI:** 10.1016/j.ymthe.2025.06.037

**Published:** 2025-06-27

**Authors:** Wolfgang Wagner

**Affiliations:** 1 Institute for Stem Cell Biology, RWTH Aachen University Medical School, 52074 Aachen, Germany; 2 Helmholtz-Institute for Biomedical Engineering, RWTH Aachen University Medical Faculty, 52074 Aachen, Germany; 3 Center for Integrated Oncology Aachen Bonn Cologne Düsseldorf (CIO ABCD), 52074 Aachen, Germany

**Keywords:** DNA methylation, 5mC, network, chromatin, lncRNA, histone code, homologous recombination

## Abstract

The epigenetic landscape governs cell fate decisions during development, aging, and disease. Despite considerable progress in the understanding of DNA methylation (DNAm), the mechanisms that orchestrate its coordinated regulation across the genome remain largely elusive. Recent breakthroughs in sequencing technologies and epigenetic editing tools enable a more comprehensive exploration of these epigenetic interactions. Regulation of DNAm seems to be organized within epigenetic networks characterized by complex feedback mechanisms acting locally, between homologous alleles, and across the entire genome. This crosstalk is facilitated by an interplay of various molecular components, including distinct variants of epigenetic writers and erasers; methylation-sensitive binding of transcription factors and other regulatory proteins that recruit DNA methyltransferases; cross-regulation between DNAm and the histone code; three-dimensional (3D) chromatin conformation; regulatory effects mediated by long non-coding RNAs (lncRNAs); and potentially by assimilation during homologous recombination events. This review explores how these diverse epigenetic mechanisms interact to collectively shape the methylome and thereby control developmental and disease processes.

## Graphical abstract

**Figure:**
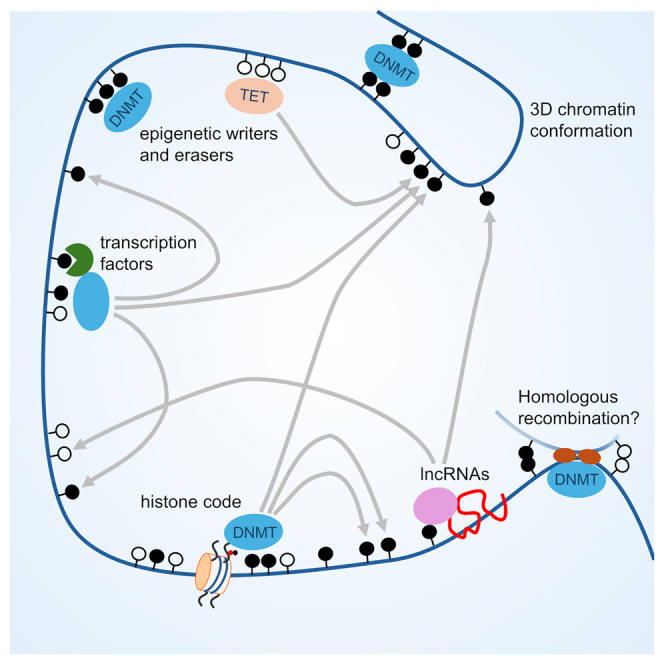

## Teaser

The DNA methylation pattern changes in very complex ways during development, aging, and disease. This review summarizes the evidence that this epigenetic landscape is regulated in a network. It discusses molecular mechanisms that may mediate this interaction and provides an outlook on mathematical approaches and therapeutic applications.

## Introduction

DNA methylation (DNAm) is an epigenetic modification that directly affects DNA conformation. The covalent attachment of methyl groups to cytosines in the DNA influences transcription factor (TF) binding, alternative splicing, chromatin looping, and interactions with various DNAm-binding proteins.^1^^,^^2^ DNAm is observed across different biological kingdoms and was initially identified for its role in bacterial genomic surveillance.^3^ In mammals, DNAm predominantly occurs at CG dinucleotides (CpGs), and it plays a crucial role in cellular differentiation. Furthermore, DNAm patterns undergo continuous changes throughout aging,^4^ and aberrant DNAm seems to contribute to development of cancer and multiple non-malignant diseases.^5^

With the development of new sequencing technologies, such as whole-genome bisulfite sequencing and Illumina BeadChip technology, thousands of DNAm profiles can now be integrated for comparative analyses.^3^ Other methods, including nanopore sequencing^6^ or PacBio Single Molecule, Real-Time (SMRT) sequencing,^7^ allow long-read measurements with simultaneous analysis of the base modifications 5-methylcytosine (5mC) and 5-hydroxymethylcytosine (5hmC). Additionally, single-cell sequencing approaches provide insights into the heterogeneity of DNAm patterns within specific cell populations, revealing how DNAm is coherently modified at the single-cell level.^8^ These technological advancements, combined with an expanding array of bioinformatics tools, have significantly enhanced our understanding of cellular differentiation.^1^^,^^3^ They have also led to the identification of new clinically relevant biomarkers—such as determining cellular composition within samples,^9^^,^^10^^,^^11^ estimating age through epigenetic clocks,^12^^,^^13^^,^^14^ or identifying malignancies based on abnormal DNAm patterns.^15^^,^^16^ The ability to accurately assess DNAm levels at single-base resolution—alongside the reproducible dynamics observed during development and disease—has made DNAm a focus of biotechnological research in recent decades.^3^

Despite this progress, it remains unclear how DNAm is coherently modified and stabilized across the entire genome. The human genome comprises approximately 28 million CpG sites,^17^ which reveal complex DNAm patterns that are highly reproducible between specific cell types—a phenomenon suggesting that non-linear feedback loops stabilize epigenetic integrity while allowing for coordinated widespread changes during differentiation processes. Traditionally, gene regulatory networks (GRNs) have been discussed to govern cell-type specification and stabilization^18^^,^^19^—it stands to reason that similar mechanisms may apply at an epigenetic level independent of specific transcriptional regulation. Several molecular parameters underlying local feedback in *cis* have been described, whereas long-range interactions in *trans* remain largely unclear.

This review first summarizes evidence that the DNAm landscape is coherently regulated and the possible mechanisms involved in this process. It then discusses the wider picture of network modeling, new opportunities by epigenetic editing, and prospects for therapeutic implications.

## Evidence for the existence of epigenetic feedback mechanisms

**Figure 1:**
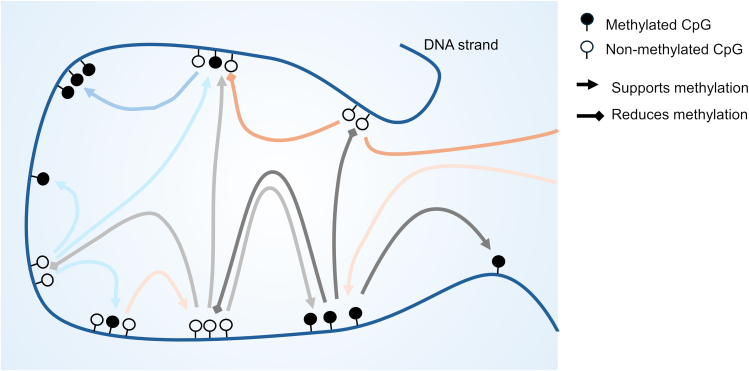
DNA methylation is regulated coherently across the genome This model illustrates the feedback mechanisms that regulate DNAm within a complex network of interactions—by mechanisms that remain to be further elucidated. The state of methylation at specific differentially methylated regions impacts gains and losses of DNAm at other genomic sites. As a result, the genome-wide DNAm patterns are co-regulated and stabilized.

The idea of epigenetic networks was already paved by Conrad Waddington when he proposed epigenetic landscapes in 1957.^20^ This model suggests that epigenetic profiles are continuously refined during development to achieve specific cell states. Coordinating such genome-wide changes requires feedback mechanisms that regulate the development in a synchronized manner (Figure 1). The mechanisms driving these long-range epigenetic interactions remain largely unknown. Validating the cross-interaction of epigenetic networks necessitates iterative testing and benchmarking of predictive models,^21^ but the technology to perturb epigenetic marks at specific genomic regions has only recently advanced.^22^ Consequently, our understanding of these intricate interactions is still in its early stages. On the other hand, the following paragraphs evidence that changes in the epigenetic landscape during cellular differentiation and aging and between different alleles are governed in a concerted manner.

### Cell-type-specific DNAm patterns are indicative for feedback mechanisms

**Figure 2:**
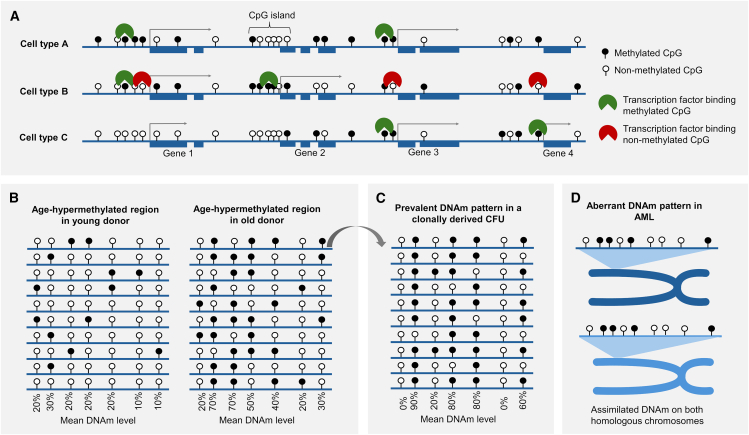
Adaptation of DNA methylation patterns in differentiation, aging, and cancer (A) This schematic illustrates cell-type-specific DNAm patterns that may reduce or enhance binding of specific transcription factors and repressors. (B) Exemplary representation of a genomic region with age-associated gain in DNAm. Notably, the DNAm at neighboring CpG sites is not necessarily co-regulated on the same DNA strand and appears to be stochastic in nature.^13^ (C) In colony-forming units (CFUs) derived from hematopoietic stem and progenitor cells, the predominant DNAm pattern predominately resembles the pattern of the CFU-initiating cell (represented here exemplarily as the top left strand in B). The deviation of this colony-initiating DNAm pattern within 14 days demonstrates the highly dynamic changes of DNAm at age-associated regions. (D) Acute myeloid leukemia (AML) is characterized by abnormal DNAm patterns. Even these aberrant patterns become homogenized between homologous chromosomes,^15^ suggesting the presence of feedback mechanisms that assimilate DNAm patterns on both alleles.

The human body comprises thousands of distinct cell types with highly specialized functions—encoding these complex differentiation processes with a single genome is one of the most remarkable achievements of biological evolution.^23^^,^^24^ Cellular differentiation is not reflected by changes in the global DNAm level but rather in cell-type-specific gains and losses of methylation at multiple specific sites in the genome (Figure 2A).^25^ Even the likelihood of methylation at these cell-type-specific CpG sites—ranging from 0% to 100% DNAm—is very similar across different samples. The number of available epigenetic cell atlases continues to grow rapidly, potentially surpassing classifications based solely on gene expression profiles.^9^^,^^25^ Moreover, comprehensive signatures comprising hundreds of CpGs can be utilized for cell-type deconvolution.^9^ The cell-type-specific DNAm patterns provide valuable biomarkers to determine the cellular composition of tissue or to identify cancer of unknown origin. In general, cell-type-specific DNAm is rather reflected by site-specific hypomethylation, whereas uniquely hypermethylated regions in specific cell types are rare.^11^^,^^26^ Furthermore, cell-type-specific DNAm is enriched for CpG islands, Polycomb targets, and binding sites of CCCTC-binding factor (CTCF), suggesting that cell-type specification is associated with new shaping of chromatin looping.^9^^,^^11^^,^^25^ It is remarkable how reproducible cell-type-specific DNAm levels at specific genomic regions are between different samples and studies. Notably, during differentiation, the cell-type-specific DNAm patterns are apparently synchronized across the entire genome. This provides clear evidence for long-range epigenetic feedback processes that co-regulate epigenetic modifications in a concerted manner.

### Dynamic and stochastic DNAm during aging

Timing is another important aspect that needs to be considered regarding epigenetic networks. This is particularly exemplified by age-associated DNAm changes, which are continuously acquired throughout life.^12^^,^^27^ Since many of these age-associated DNAm changes follow a nearly linear trajectory, epigenetic signatures can be used for age predictions.^28^^,^^29^ Such "epigenetic clocks" have gained widespread application for estimating chronological age in forensic contexts or providing insights into biological aging processes.^14^^,^^30^ In fact, it has been demonstrated that epigenetic clocks are associated with all-cause mortality and various diseases—hence, depending on the signature used, they reflect aspects of the individual aging process.^31^^,^^32^^,^^33^ The underlying mechanism that drives epigenetic clocks is not yet fully understood,^28^ but there is some insight into their dynamics.

Several recent studies indicated that the process by which age-associated DNAm is acquired is quasi-stochastic.^34^ Simulations based on DNAm profiles of sorted immune cells suggested that up to 90% of the accuracy underpinning epigenetic clocks could be driven by a stochastic process, whereas parameters that are rather indicative for biological aging are more reflected by non-stochastic means.^35^ Importantly, the assumption of stochastic components is also compatible with epigenetic networks—feedback mechanisms in *trans* may modulate the stochastic likeliness to gain or lose methylation. While empirical simulations or bulk analysis of many cells provides DNAm levels ranging from 0% to 100%, the CpG sites on individual DNA strands can only have two states: methylated or non-methylated. Thus, the gradual DNAm changes during aging or differentiation follow a probability distribution at each individual CpG site (Figure 2B).^13^^,^^36^ Yet, the question remains why only specific CpG sites gain or lose DNAm with time and how these stochastic changes are governed to follow a highly consistent trajectory over the many years of human life. This may be favored by specific histone modifications, chromatin structure, or *trans*-regulatory feedback processes.

In addition to the stochastic processes at individual CpG sites, there seems to be also stochastic variation between neighboring CpGs. Analysis of DNAm patterns on individual DNA strands demonstrates that neighboring CpGs are not coherently modified but rather fluctuate in their DNAm level independent from neighboring sites^13^^,^^36^—a finding referred to as epiallelic entropy.^37^ This may be counterintuitive, as binding of DNA methyltransferases (DNMTs) to a specific genomic region should also impact neighboring CpGs—in analogy to the covalent histone modifications that are similarly modified on nearby nucleosomes.^38^ Apparently, the epigenetic writers are not affecting the neighboring DNA sequences of a target site, or the process is more dynamic than generally anticipated. Within-sample heterogeneity scores provide alternative tools for estimating variance in DNAm patterns and may provide insight into disease-associated mechanisms not captured by established statistics.^39^ The sum of residuals of individual age-associated CpGs, determined by a variation score, may better reflect aspects of biological aging than conventional epigenetic clocks.^29^ To gain further insight into the dynamics of age-associated DNAm, we have recently used amplicon sequencing for colony-forming units (CFUs) of hematopoietic stem and progenitor cells.^40^^,^^41^ Since each of the CFUs is clonally derived and captures the epigenetic makeup of the colony-initiating cell, such analysis can provide insight into the dynamics of how DNAm patterns diversify *in vitro*. After 14 days, there was still a dominant DNAm pattern at age-associated regions, while there was already divergence among the individual reads (Figure 2C)—a testament to the dynamic nature of DNAm patterns. If age-associated DNAm fluctuates at very high rates in a quasi-stochastic and highly dynamic manner, how can this be controlled over the lifetime? One possible explanation might be that feedback mechanisms within an epigenetic network modulate and stabilize these changes throughout life.^42^

### DNAm is coordinated across different alleles

Further evidence for epigenetic networks comes from the observation that DNAm is co-regulated at different sites in the genome—and even between different alleles. The DNAm pattern is predominantly identical across homologous chromosomes. This overall symmetry of DNAm profiles on both alleles suggests epigenetic crosstalk between homologous chromosomes. Notably, even aberrant DNAm patterns in acute myeloid leukemia (AML) revealed very similar aberrant DNAm patterns at neighboring CpGs in amplicon bisulfite sequencing data.^15^ Since these aberrant patterns are not acquired during normal development, it might be anticipated that defective DNAm patterns during disease development occur only on individual alleles. In contrast, there were significant and patient-specific changes in DNAm patterns at first diagnosis, remission, and relapse in AML—and these DNAm patterns were always almost identical at both alleles (Figure 2D).^15^

Allele-specific methylation (ASM) occurs when DNAm patterns have asymmetry among alleles.^43^ This was first identified at imprinted loci, where ASM is established in either female or male germlines, resulting in parent-of-origin-specific gene expression.^44^ Thus, genomic imprinting ensures monoallelic expression of imprinted genes, and disruption can result in disorders such as Prader-Willi syndrome or Beckwith-Wiedemann syndrome.^45^ About 100 genes have been verified as imprinted in the human genome, while the real number may be considerably higher.^46^ Furthermore, ASM also occurs at other sites in the genome outside of imprinted loci.^47^ Studying genome-wide patterns of ASM in humans is enabled by deep sequencing technologies, where single-nucleotide variations (SNVs) are used to discern the two different alleles,^48^ which is even applicable on the single-cell level.^49^ For example, large regions with ASM have been mapped to procadherin and *HOX* gene clusters, suggesting the differential DNAm is relevant for the complex transcription regulation at these important developmental regions.^48^ Furthermore, ASM is increased in some cancers^50^ and can serve as an effective tumor marker.^51^ In general, it appears that the majority of ASM is associated with genetic variation in *cis*.^46^ Heterozygous SNVs seem to disrupt the methylation pattern nearby.^52^

While the above examples underscore the importance of ASM, the overall similarity of DNAm profiles on homologous chromosomes indicates the existence of a *trans*-acting molecular process with sequence-specific feedback that seems to be impaired by nearby SNVs—which may provide further hints into the potentially underlying mechanisms of epigenetic feedback loops.

## Molecular mechanisms that mediate epigenetic networks

To establish epigenetic networks that coordinate and stabilize DNAm patterns across the genome, over time, and between different alleles, it is essential to have molecular mechanisms that mediate this interplay. Although the precise nature of these *trans*-acting epigenetic mechanisms is still not fully understood, the following mechanisms seem to be involved in shaping of the epigenetic landscape (Table 1).

**Table 1:** Molecular players that shape the epigenetic landscape

Molecular mechanism	Examples	Regulatory function within the network
Epigenetic writers	DNA methyltransferases: DNMT1, DNMT3A, DNMT3B, and DNMT3L (no DNMT activity)	• DNMTs have binding preferences to sequence motives • different splice variants of DNMTs can have different binding preferences and protein interactions • mutations in DNMTs can impact activity and binding
Epigenetic erasers	ten-eleven translocation proteins: TET1, TET2, and TET3	• TETs have binding preferences to sequence motives • different splice variants of TETs can have different binding preferences • mutations in TETs can impact activity and binding
Epigenetic readers	transcription factors (TFs), and methyl-CpG binding proteins (MBDs)	• binding preferences for methylated or non-methylated sequences • can recruit repressors, epigenetic writers, and epigenetic erasers
Histone code	a wide range of histone modifications; they are mediated, for example, by Polycomb repressive complexes PRC1 and PRC2	• changes chromatin conformation and accessibility to epigenetic writers • DNMTs bind to specific histone modifications • PRCs interact with DNMTs
3D chromatin conformation	topologically associated domains (TADs) mediated, e.g., by CTCF	• changes chromatin accessibility • brings epigenetic modulators into vicinity for coherent modifications • lamina-associated domains foster repressive chromatin environment
Long non-coding RNAs	e.g., *HOTAIR*, *ANRIL*, *XIST*, and *NEAT1*	• may form triple helix with target regions • direct the chromatin-modifying complexes and epigenetic writers to specific sites in the genome
Homologous recombination	mediated by recombinase, e.g., RAD51	• hypothetical role for adaptation of DNA methylation pattern between homologous chromosomes and in *trans* on related sequences (remains to be demonstrated)

### Different targets of diverse epigenetic writers and erasers

**Figure 3:**
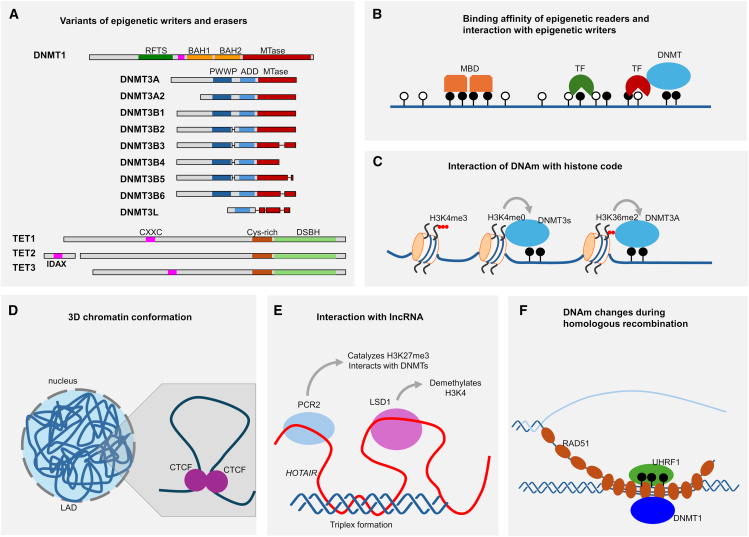
Mechanisms contributing to coordinated modifications of DNAm profiles (A) Schematic representation of various isoforms of DNA methyltransferases (DNMTs) and ten-eleven translocation (TET) methylcytosine dioxygenases.^53^^,^^54^^,^^55^ The C terminus of DNMTs has the catalytic methyltransferase (MTase) domain. Variation exists, particularly in the regulatory regions that include the replication foci-targeting sequence (RFTS); a DNA-binding zinc finger cysteine-X-X-cysteine (CXXC) implicated in binding CpG-containing DNA sequences; bromo-adjacent homology domains (BAHs); proline-tryptophan-tryptophan-proline domain (PWWP); and ATRX-Dnmt3-Dnmt3L domain (ADD) that recognizes histone H3 tail unmethylated at lysine-4. All three TET members feature a double-stranded β-helix domain (DSBH) at the C terminus that is responsible for iron binding and is associated with the oxygenase activity, a cysteine-rich region, and a CXXC domain thought to recognize unmethylated CpGs.^56^ For TET2, the function of the CXXC domain appears to be compensated by the neighboring gene: inhibition of the Dvl and AXin complex (*IDAX*).^57^ (B) Methyl-CpG binding domain proteins (MBDs) bind to symmetrically methylated CpGs, leading to gene expression repression. Transcription factors (TFs) often have a binding preference for either methylated or non-methylated CpGs, with some directly interacting with DNMTs. (C) Two examples illustrating how histone modifications influence DNAm: unmethylated H3K4 can bind to the ADD domain of DNMT3s, while H3K36me2 interacts with the PWWP domain of DNMT3A. (D) The three-dimensional chromatin conformation and lamina-associated domains (LADs) are tightly regulated. CCCTC-binding factor (CTCF), a methylation-sensitive transcription factor, plays a key role in forming chromatin loops. (E) The long non-coding RNA *HOTAIR* binds DNA via triplex formation and interacts with the Polycomb repressive complex 2 (PRC2) as well as lysine-specific demethylase 1 (LSD1).^58^ (F) RAD51 coats single-stranded DNA and scans the genome for homology repair. RAD51 inhibits ubiquitin-like with PHD and RING finger domains 1 (UHRF1) and recruits and stabilizes DNMT1.^59^

A fundamental approach to establishing specific DNAm patterns involves utilizing various proteins that directly modify these patterns in distinct ways. Indeed, DNMTs exhibit functional diversity through alternative splicing, resulting in varied enzymatic activities and binding specificities (Figure 3A).^60^^,^^61^^,^^62^ For example, DNMT1, commonly referred to as the "maintenance" methyltransferase, exists in three splice variants: DNMT1s is expressed in somatic cells, DNMT1p is found exclusively in pachytene spermatocytes, and DNMT1o is specifically expressed in oocytes and preimplantation embryos.^63^ DNMT3A and DNMT3B, classified as *de novo* methyltransferases,^64^ are also subject to extensive tissue- and developmental-stage-specific alternative splicing.^60^ The full-length isoform DNMT3A1 is broadly expressed across adult tissues, whereas the truncated isoform DNMT3A2 lacks a regulatory domain and is predominantly found in embryonic stem cells (ESCs).^65^ Numerous studies have explored the functions of specific DNMT3A splice variants by targeting specific transcripts or genetic mutations.^66^^,^^67^^,^^68^ DNMT3B is even expressed in more than 30 isoforms, with alternative splicing occurring in both the catalytic and regulatory domains.^69^^,^^70^ The full-length transcript DNMT3B1 is characteristically highly expressed in naive ESCs. DNMT3-like (DNMT3L) has no methyltransferase activity, but it interacts with DNMT3A and DNMT3B to simulate their enzymatic activities and target them to chromatin.^71^ Interestingly, two DNMT3B isoforms—DNMT3B3 and DNMT3B6—share the same deletion of the catalytic domain as DNMT3L and demonstrate comparable potency in stimulating other DNMTs *in vitro*.^72^

While DNAm is generally stable, it can be reversed through ten-eleven translocation (TET) family proteins or via passive demethylation during replication.^56^ TET proteins catalyze the oxidation of 5mC, facilitating active DNA demethylation via an intermediate product, 5hmC, which resembles another important epigenetic mark with so far largely unknown physiological significance.^1^ Other oxidation products of 5mC, such as 5-formylcytosine (5fC) and 5-carboxylcytosine (5caC), can be re-converted into cytosine by thymine DNA glycosylase (TDG) with base excision repair.^73^

The TET family comprises the dioxygenases TET1, TET2, and TET3. Furthermore, TET1 exists in an N-terminal truncated form (TET1s), which overall seems to have reduced DNA binding affinity and weaker demethylation activity as compared to the full-length transcript (TET1e).^73^ Each TET isoform has unique expression patterns.^56^^,^^73^^,^^74^ For instance, TET3 plays a pivotal role in the active demethylation of the paternal genome post-fertilization, while TET1 and TET2 have broader roles in maintaining epigenetic balance.^53^ Recently, it has been shown that TET-mediated demethylation occurs only when DNMTs are absent, indicating that there is competition between epigenetic writers and erasers at individual CpGs.^75^ Specific mutations in DNMT3A and TET enzymes are frequently observed in various malignancies,^76^ whereas other mutations are enriched in clonal hematopoiesis of indeterminate potential (CHIP),^77^ supporting the relevance of these mutational variants for hematopoietic development. Thus, there is a broad arsenal of epigenetic writers and erasers that can modify the epigenetic network in manifold ways.

In fact, DNMT3A and DNMT3B have moderate binding preferences to specific sequence motives at the flanks of CpG sites, which may play a crucial role in their diverse function during development and pathogenesis.^78^ It has also been demonstrated that such motives are associated with methylated and unmethylated CpGs, which coincides with their differential binding affinity to DNMT3A and TET1.^79^ Furthermore, disruption of these motifs by mutation affected DNAm patterns next to the target site, indicating that the DNA sequence has an immediate impact on the binding of epigenetic writers and hence on the local DNAm dynamics.^79^ While these sequence binding preferences were highly significant overall, it appears that genomic targeting of DNMTs and TETs is rather regulated by additional proteins that interact with the regulatory N-terminal regions, including DNMT3L.^78^

In contrast to other mechanisms that act on DNAm in *trans*, the specific molecular sequels of different variants of epigenetic writers are relatively well understood, particularly in pluripotent cells and hematopoietic differentiation.^68^^,^^80^ Although different isoforms of both DNMTs and TETs serve distinct functions throughout development and disease progression, it is evident that their composition alone cannot fully explain the highly diverse DNAm landscapes.

### Epigenetic readers can modulate the epigenetic landscape

Theoretically, the DNAm network can be effectively controlled by a small number of DNA-binding proteins that recruit epigenetic writers and erasers to specific sites in the genome. Since the binding of DNA-binding proteins is influenced by DNAm, this mechanism can mediate feedback mechanisms. The addition of methyl groups to DNA alters the conformational dynamics of the double helix.^81^ These structural changes significantly affect the binding of many TFs and regulatory proteins, which are collectively referred to as epigenetic readers. The methylation status of TF binding sequences has a direct impact on their binding affinity, particularly when those motifs include CpG sites.^82^ Computational predictions can estimate how 5mC influences DNA shape and its subsequent impact on TF binding.^81^^,^^83^ For instance, specificity protein 1 (SP1) typically binds to hypomethylated GC-rich promoter regions and plays a role in inhibiting DNMTs.^84^ The proto-oncogene c-MYC preferentially binds to non-methylated E-box motifs, while it has also been shown to interact with DNMT3A to methylate target sequences.^85^^,^^86^ In contrast, the TFs ZFP57 and C/EBPβ have a binding preference for methylated E-box motifs (Figure 3B).^83^

Moreover, various proteins bind to methylated DNA even without a preference for specific DNA sequences, such as methyl-CpG binding domain proteins (MBDs), including MeCP1 and MeCP2.^87^^,^^88^ These proteins recognize and bind to methylated genomic regions, leading to either transcriptional repression or activation depending on the cellular context.^89^ Additionally, they can recruit co-repressors or chromatin remodeling complexes that further modify local chromatin structure and influence accessibility for specific TFs.

While the interaction of TFs with DNMTs and histone-modifying proteins has been demonstrated, it remains to be proven whether this plays an essential role in the concerted epigenetic regulations in *trans*. Importantly, MBDs and TFs can also interfere with the histone code and thereby further modulate the epigenetic landscape.

### The histone code modulates local DNAm patterns

Chromatin is organized into multiple structural layers with DNA wrapped around nucleosomes that consist of octameric complexes formed by histone proteins (H2A, H2B, H3, and H4), facilitating the compaction of DNA. Histone H1 plays a crucial role in linking nucleosomes together, thereby stabilizing higher-order chromatin structures. While histone proteins are expressed in various isoforms, the intricate array of post-translational modifications known as the histone code has an even more significant influence on DNA binding and chromatin accessibility.^90^ The tails of histone proteins can undergo a variety of post-translational modifications, including methylation, acetylation, phosphorylation, and ubiquitination at specific amino acid residues. Thus, the histone code provides another layer of epigenetic complexity, which seems to be tightly interwoven with the methylome.

For instance, trimethylation of histone H3 at lysine 4 (H3K4me3) is typically associated with active promoters and correlates with low levels of DNAm. Conversely, when H3K4 is unmethylated, it can bind to the ATRX-Dnmt3-Dnmt3L (ADD) domain of DNMT3A or DNMT3B, thereby activating their methyltransferase domains (Figure 3C).^1^^,^^91^ In contrast, H3K36 dimethylation (H3K36me2), deposited by the nuclear receptor binding SET domain protein 1 (NSD1), corresponds to elevated intergenic levels of DNAm. The proline-tryptophan-tryptophan-proline domain (PWWP)—conserved among *de novo* methyltransferases—interacts with both H3K36me2 and H3K36me3 and is essential for the proper localization of DNMT3A.^92^^,^^93^ In murine oocytes, simultaneous depletion of H3K36me2 and H3K36me3 has been shown to result in global hypomethylation comparable to that observed with DNMT3A depletion.^94^ Lower DNAm levels facilitate the binding of the Polycomb repressive complex 2 (PRC2), which catalyzes the repressive histone mark H3K27me3 and can interact with DNMTs as well.^1^^,^^95^ Additionally, DNMT1 interacts with marks such as H3K9me3^96^ and H4K20me3.^97^ The presence of H3K9me3 recruits proteins, like heterochromatin protein 1 (HP1), that promote chromatin compaction while also enhancing the recruitment of DNMTs, leading to increased DNAm in adjacent regions.^98^^,^^99^ Active marks such as acetylated lysines on histone 3 (H3Kac) are known to inhibit DNMT activity, fostering an open chromatin state that is conducive to gene expression.^100^ Interestingly, it has also been suggested that Dnmt1 may be associated with histone deacetylase (HDAC) activity *in vivo*.^101^ This intricate interplay between the histone code and DNAm highlights a broader regulatory network where various epigenetic modifications collaboratively shape cellular identity and function. However, despite this tight interaction between different epigenetic mechanisms, questions remain about how these modifications facilitate the long-range interactions within an epigenetic network.

### The impact of 3D chromatin conformation on the DNAm landscape

The epigenetic network can also be perceived by the spatial organization of the genome in the nucleus. The three-dimensional (3D) organization of the genome is specific to each cell type and is crucial for maintaining the functional integrity of nuclear architecture. Various chromatin conformation capture techniques reveal interactions of genomic regions by cross-linking chromatin, digesting it with restriction enzymes, and then ligating the fragments—such as the genome-wide Hi-C sequencing approach.^102^ Comprehensive investigations, including single-cell analysis, have highlighted the importance of nuclear organization for cell-type specificity and epigenetic configurations.^103^^,^^104^ The spatial conformation of chromatin influences how accessible DNA is to epigenetic writers and erasers.^105^ For example, regions tightly packed within heterochromatin are generally less accessible to DNMTs, resulting in lower DNAm levels compared to more accessible euchromatic regions.^106^

The compartmentalization of chromatin can facilitate regulatory hubs that enhance interactions between TFs, creating transcription factories with discrete sites of gene expression^107^ while coordinating the activity of epigenetic modifiers.^108^ Changes in DNAm patterns can disrupt topologically associated domains (TADs) and alter chromatin structure. A key architectural protein, CTCF, binds to specific DNA sequences and plays a pivotal role in organizing the 3D structure of the genome.^109^ As an insulator, CTCF prevents inappropriate interactions between enhancers and promoters, thereby ensuring proper gene regulation. Importantly, CTCF binding is sensitive to DNAm; hypermethylation at CTCF binding sites can inhibit its ability to bind effectively. This disruption may lead to altered chromatin loops and changes in enhancer-promoter interactions, ultimately impacting gene expression profiles (Figure 3D).^110^^,^^111^ Furthermore, lamina-associated domains (LADs) are genomic regions anchored to the nuclear lamina—a dense fibrillar network located just beneath the inner nuclear membrane.^112^^,^^113^ This association fosters a repressive chromatin environment characterized by lower levels of DNAm and repressive histone modifications such as H3K9me2/3 and H3K27me3.^114^^,^^115^^,^^116^

Thus, chromatin conformation plays a central role in shaping the complex regulatory network that governs the methylome. However, at this point, it is hardly possible to precisely control chromatin conformation for functional analysis. Furthermore, the 3D interaction network is so complex and intertwined with other epigenetic mechanisms that we can currently only estimate their relationships.

### Long non-coding RNAs direct epigenetic writers to specific sites in the genome

Another player that may be involved in the concerted regulation of the DNAm landscape is the role of long non-coding RNAs (lncRNAs). In recent years, lncRNAs have emerged as pivotal regulators of gene expression, and their role in modulating DNAm profiles has garnered significant attention.^117^ Unlike protein-coding genes, the function of lncRNAs appears to be primarily characterized by their ability to interact with various molecular players, including chromatin-modifying complexes and TFs.

One prominent example is the lncRNA HOX transcript antisense RNA (*HOTAIR)*, which is transcribed from the HOXC locus.^118^ *HOTAIR* has been shown to interact with PRC2 and lysine-specific demethylase 1 (LSD1), facilitating their recruitment to target genes.^119^^,^^120^ Notably, targeting of *HOTAIR* to specific sites in the genome seems to be mediated via triplex-helix formation with target regions.^58^ Chromatin isolation by RNA purification (ChIRP) sequencing identified 832 *HOTAIR* occupancy sites in a breast cancer cell line, indicating that *HOTAIR* target regions are focal, sequence specific, and numerous.^121^ This illustrates how a specific lncRNA can guide epigenetic modifiers to genomic sites, leading to the trimethylation of H3K27 or the demethylation of H3K4, respectively—ultimately resulting in the epigenetic silencing of target regions (Figure 3E).

Similarly, the antisense non-coding RNA in the INK locus (*ANRIL*) interacts with PRC1 and PRC2 complexes to facilitate the trimethylation of H3K27. Another significant lncRNA is X-inactivation-specific transcript (*XIST*), which plays a vital role in X chromosome inactivation in female mammals.^122^^,^^123^ This process involves ASM on the inactive X chromosome by DNMT3A/B.^124^ Moreover, nuclear paraspeckle assembly transcript 1 (*NEAT1*) has been shown to influence DNAm dynamics during cellular differentiation. In pluripotent stem cells, *NEAT1* can recruit TET enzymes and PRC2 to promote epigenetic changes essential for maintaining pluripotency.^125^

The examples of *HOTAIR*, *ANRIL*, *XIST*, and *NEAT1* highlight how lncRNAs can play a pivotal role in shaping DNAm profiles by directing chromatin-modifying complexes to specific sites within the genome. Since the expression of lncRNAs is also regulated by epigenetic modifications, they may facilitate feedback loops within the epigenetic network.

### The potential role of homologous recombination in DNAm changes

Homologous recombination (HR) facilitates the exchange of genetic material between homologous chromosomes, playing a crucial role in repairing DNA double-strand breaks and promoting genetic variability throughout evolution.^126^ Although experimental evidence linking HR to epigenetic adaptations remains limited, this discussion will explore its potential significance for orchestrating coordinated changes in the methylome.

At the core of HR is a DNA recombinase that coats single-stranded DNA (ssDNA) to form a nucleoprotein filament. This filament must then interact with double-stranded DNA (dsDNA) to locate and pair with homologous DNA, necessitating a thorough scanning of the genome for sequence complementarity.^127^ The first identified recombinase was the bacterial recombinase A (RecA),^128^ while in eukaryotes, the RAD51 recombinase (RAD51) serves as its ortholog, executing essential steps such as strand invasion, homology search on sister chromatids, and strand exchange.^129^^,^^130^ For an efficient homology search, dsDNA must be transiently bound and quickly released when mismatched sequences are encountered. This dynamic process involves the ATPase activity of RAD51 and potentially other cofactors.^129^ Apparently, little attention has been given to how DNAm patterns might influence this homology search—however, this might be anticipated given that DNAm affects DNA-DNA binding affinity, e.g., with regard to melting and hybridization temperatures.^81^^,^^131^

Recent studies have revealed that RAD51 has functions extending beyond HR.^132^ Notably, RAD51 has been shown to interact with and inhibit ubiquitin-like with PHD and RING finger domains 1 (UHRF1), a methyl-binding protein that preferentially binds to hemi-methylated DNA and recruits DNMT1.^59^ Deficiency in RAD51 results in excessive ubiquitination and degradation of DNMT1, leading to a significant loss of global DNAm.^132^ Additionally, RAD51 assists UHRF1 in monoubiquitinating histone H3, generating signals that recruit DNMT1. Disruption of the interaction between RAD51 and histone H3 reduces DNMT1 recruitment, ultimately impairing the maintenance methylation of genomic DNA.^132^ Given that RAD51 may facilitate direct contact between differentially methylated DNA strands—allowing them to be recognized as hemi-methylated by DNMT1—I hypothesize that the HR-mediated interactions between alleles could play a crucial role in epigenetic adaptation among homologous chromosomes (Figure 3F).

The significance of HR in the adaptation of DNAm patterns is further underscored by the observation that homology-directed repair of a DNA double-strand break recruits DNMTs and entails methylation of the repaired region.^133^ A method known as homology-assisted repair-dependent epigenetic engineering (HARDEN)^134^ leverages endogenous DNA double-strand break repair pathways to facilitate targeted DNAm through homology-directed repair, utilizing an *in*-*vitro*-methylated exogenous repair template. Remarkably, it has been suggested that HARDEN could achieve higher levels of targeted DNAm compared to dCas9-DNMT3A fusion proteins.^134^ While this experimental approach may require further validation, it supports the hypothesis that HR plays a crucial role in assimilating the DNAm patterns.

Various genetic disorders are characterized by defects in DNA repair mechanisms and associated with the HR pathway—such as Fanconi anemia,^135^ Bloom syndrome,^136^ and ataxia-telangiectasia^137^—reveal aberrant DNAm profiles or accelerated epigenetic aging. Moreover, studies involving single breaks in the DNA of mouse and human cells have shown that these genomic regions often experience gains or losses of DNA.^138^ A comprehensive analysis of multiple datasets has recently revealed that CpG mutations not only correlate with changes in methylation at the mutated site but also lead to extensive remodeling of the methylome up to ±10 kb away.^139^ Various mechanisms have been proposed regarding how somatic mutations may be associated with DNAm at neighboring sequences—the deamination might be triggered in a hypermethylated environment, mutations might affect the binding sites of epigenetic writers, or mutations and DNAm might both be a result of doublestrand breaks with defective repair mechanisms during HR.^139^^,^^140^

It is conceivable that the RAD51 protein machinery not only mediates localized changes in DNAm during HR through interactions between strictly complementary regions on homologous chromosomes but also exerts effects across the entire genome in *trans*. As RAD51 scans the entire genome for homologous sequences, it may stall at other related sequences. The genome-wide interactions facilitated by such DNA-DNA bindings could then contribute to the complex regulation within the broader network of DNAm.

## Epigenetic editing provides further insight into epigenetic networks

To validate the existence of epigenetic networks and explore feedback mechanisms, it is important to experimentally perturb the DNAm pattern in a controlled manner. Recent advancements in epigenetic editing technologies have enabled focused manipulation of DNAm, offering powerful tools to investigate the functional relevance of specific epigenetic marks.^141^ These approaches often utilize CRISPR-mediated systems, where a catalytically inactive Cas9 (dCas9) is fused with epigenetic effectors such as DNMT3A (for methylation) or TET1 (for demethylation).^142^ At many target regions, DNMT3A-mediated epigenome editing has been shown to be rather transient and was lost within a few days or weeks,^143^ but it has also been demonstrated that epigenetic editing of DNAm can be stable for several months and even during myeloid differentiation in a murine transplantation model.^144^^,^^145^ Furthermore, fusion proteins have been developed for more sustained manipulations, such as “CRISPRoff,” which combines DNMT3A/3L with a transcriptionally repressive Krüppel-associated box (KRAB) domain.^146^^,^^147^ These tools can be used to determine the impact of site-specific DNAm on gene expression and splicing events. They can also provide insight into dynamics and the heterogeneity of DNAm patterns. For instance, allele-specific DNAm editing at gene promoters containing SNVs offers therapeutic potential for correcting aberrant imprinting in disorders.^148^^,^^149^

**Figure 4:**
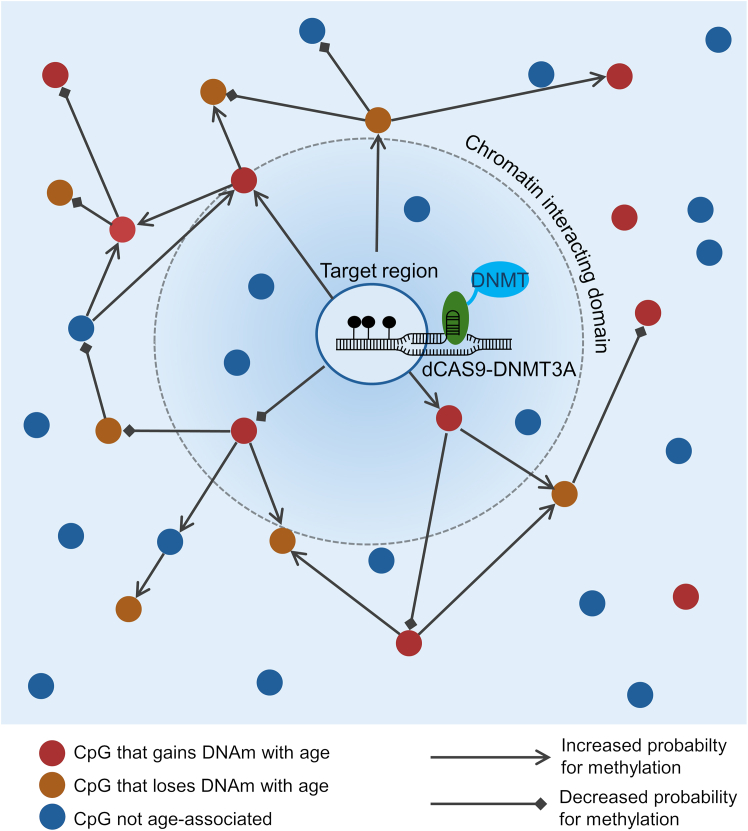
Hypothetical model of bystander modifications upon epigenetic editing Epigenetic editing with CRISPR-DNMT3A not only evokes on-site modifications but has also many off-target effects.^151^ We have recently demonstrated that targeting genomic regions with age-associated DNAm entails such off-target effects that are highly reproducible, guide RNA specific, not related to predicted target sites, and significantly enriched at other CpGs that either gain or lose DNAm with age.^42^ Furthermore, there was evidence that these bystander modifications are enriched at chromatin interacting regions. This points toward an epigenetic network in which differentially methylated regions communicate with one another.^42^

Although dCas9-methyltransferases have been shown to have high specificity for on-target modifications, there was also a surprisingly high number of off-target effects.^150^^,^^151^ Such off-target sites are observed across the entire genome. They are often single guide RNA (sgRNA) dependent and have been shown to be highly reproducible in independent replicas.^42^^,^^144^ Thus, it may be conceivable that at least some of these off-targets are due to co-regulation within the epigenetic network—and might, therefore, rather be considered as bystander modifications, which are not directly targeted by the sgRNA but potentially resemble downstream modifications due to the change at the target region. Recently, our team targeted age-associated CpG sites to explore the impact of epigenetic editing on epigenetic clocks.^42^ These edits were particularly stable in regions that gain methylation with age, while we also observed many highly reproducible off-target effects. Surprisingly, these modifications were highly enriched at other age-associated CpGs—that either gain or lose DNAm with age—and this association was increased with the age relatedness of the genomic region. Moreover, off-target effects were enriched at open chromatin regions and interacting chromatin domains, suggesting the presence of an epigenetic network that facilitates communication between differentially methylated regions (Figure 4). This phenomenon hints at broader epigenetic dynamics where interactive networks may influence not only age-associated DNAm patterns but also developmental and disease-associated processes.

## Biological networks: The wider picture

Biological networks comprise many different molecular entities that are regulated collectively—yet the mathematical approaches to understanding this interaction are often related. Analysis of biological networks is based on bioinformatic and mathematical frameworks that usually involve data collection, building of the network, testing the network with an independent dataset, visualization, and biological interpretation.^152^ Fundamental principles connect network structures to biological functions.^153^ Biological networks are typically characterized by several key features, including complexity, hierarchical organization, robustness, and dynamic adaptivity. By modeling such interactions, it is possible to predict how perturbations affect the system, facilitating advancements in drug development and personalized medicine—a development that is further revolutionized by artificial intelligence (AI).^154^^,^^155^

Multiple statistical and numerical methods are available to perform regression analysis on network structures, to assess similarities and differences between networks at different stages, and to identify regulatory hubs.^156^^,^^157^ Co-expression networks are widely used, for example, in RNA sequencing data, to identify modules of co-expressed genes that are members of the same biological pathways.^158^ They can identify modules of highly correlated molecular features, relate these features to one another or to external sample traits, and determine module membership measures.^159^ So-called parenclitic networks establish links between parameters (nodes) without any *a-priori* knowledge of their interactions by using residual distances from linear regression models constructed between every pair of parameters as edge-weights.^160^^,^^161^ Furthermore, machine learning approaches can predict interactions and regulatory relationships, even if the underlying rationale remains unclear. Thus, there are many mathematical and computational approaches for biological networks, and a single approach may not reflect the entire structure.^162^

Integration of diverse data types—such as transcriptomic, genomic, proteomic, and metabolomic datasets—offers a holistic view of cellular functions. For example, GRNs integrate genes, TFs, and other molecules that regulate gene expression.^18^^,^^19^ GRNs can also include interaction with microRNAs (miRNAs),^163^ lncRNAs,^164^ and alternative splicing.^165^ Other networks involve metabolism, protein interactions, or regulatory functions.^166^ It is not trivial to correlate DNAm with gene expression, as there are many CpGs in each gene, which oppose simple comparisons. A better understanding of how GRNs interact with epigenetic networks may allow reliable predictions of how specific DNAm patterns affect gene expression or splicing. Either way, epigenetic regulation impacts GRNs, and vice versa. In this context, epigenetic networks can be considered an additional layer of GRNs. However, since differential gene expression often arises because of epigenetic modifications and because epigenetic networks also modulate genomic regions outside of genes, these networks may also warrant independent focus.

## Epigenetic networks for clinical application

Understanding biological networks, particularly disease-specific molecular networks, holds immense potential for enhancing the diagnosis, prognosis, and treatment of various diseases.^167^^,^^168^ Integrating omics datasets allows the identification of relevant pathways or disease modules that can be utilized for disease stratification and personalized treatment regimens.^169^ To date, network-based medicine has primarily focused on protein-protein interactions, GRNs, and metabolomic networks.^170^ However, the incorporation of DNAm profiles into these networks has been shown to be effective in identifying altered pathways,^171^ although DNAm patterns have received comparatively less attention than other aspects of biological systems.

One promising application of epigenetic networks is the establishment of biomarkers. Unlike conventional approaches to epigenetic biomarker development, this strategy assumes that the regulatory mechanisms governing the epigenetic landscape are altered in ways that result in disease-specific DNAm modules. Particularly in cancer, which usually exhibits marked alterations in the epigenetic landscape, it appears likely that epigenetic networks are rewired in their entirety due to aberrant regulatory mechanisms. For instance, pancreatic ductal adenocarcinoma has been studied through a multi-parametric integrative analysis of transcriptome data, histone modifications, and methylation patterns to identify subtype-specific biomarkers.^172^ Furthermore, algorithms have been established that might identify epigenetic modules for diseases, for example, to predict breast cancer subtypes or to estimate the survival time of patients.^173^ Despite these promising early findings regarding network-based integration of DNAm, clinical translation is so far still lacking.^168^ This may partly be attributed to the abovementioned lack of mechanistic understanding of how the DNAm network is coherently regulated. Another difficulty arises from cellular heterogeneity, which notorious impacts DNAm profiles. Unless epigenetic profiles are conducted on specific purified cell types of either a healthy or malignant phenotype, identified disease-specific modules may not accurately reflect alterations within malignant cells but rather indicate changes in cellular composition.^41^ Furthermore, genome-wide analyses of DNAm profiles can be expensive, and the methods need to be validated and approved for clinical application. In this context, targeted analysis focusing on specific CpG sites might offer advantages,^174^ and therefore, it may be important to boil the network analysis down to a few epigenetic hubs that are characteristic of the disease-associated network alterations.

The use of epigenetic network analysis for developing targeted therapeutic strategies might be an even bigger challenge. Several drugs that modulate the epigenome—referred to as "epi-drugs"—have received clinical approval. These include azacitidine and decitabine, which are nucleoside analogs that inhibit DNMTs and are used for the treatment of various myeloid malignancies, including AML.^168^ Furthermore, vorinostat, romidepsin, belinostat, and panobiostat are HDAC inhibitors that are used for the treatment of T cell lymphoma and multiple myeloma.^168^ However, these medications do not specifically target components within the epigenetic network. While epi-drugs can positively influence dysregulated epigenetic networks, adopting a more holistic approach aimed at repairing these networks in a tailored manner would be intriguing. By deepening our understanding of how these networks function, we may identify new therapeutic options, e.g., by selectively modulating the expression of specific isoforms of epigenetic writers or readers and lncRNAs or even employing techniques for precise epigenetic editing to correct aberrant DNAm patterns.

## Conclusions

Over the past few decades, significant progress has been made in elucidating the regulatory mechanisms that shape the epigenetic landscape.^3^ However, most studies have primarily focused on site-specific DNAm in a rather descriptive manner, leaving gaps in our understanding of how these epigenetic profiles are harmonized across the genome. It is evident that this coordinated process involves multiple complex regulatory layers: (1) differential expression of isoforms of epigenetic writers; (2) targeting of epigenetic writers to specific sites in the genome by DNA-binding proteins; (3) histone modifications that modulate DNAm, and vice versa; (4) modulation of DNAm by the 3D chromatin conformation; and (5) lncRNAs that recruit epigenetic writers to specific sites in the genome. Furthermore, the protein machinery involved in HR might contribute to long-range adaptations of DNAm profiles. This multilayered regulatory network likely surpasses the complexity of traditional GRNs, which are largely focused on the regulation of gene expression levels. However, it is important to realize that we are only just beginning to understand how these long-range interactions might possibly act. It is hardly clear which of the above mechanisms plays the most important role and how they really impact the epigenetic landscape. The datasets already available that can shed light on epigenetic regulation in *trans* are still quite limited. Moreover, their integration is complicated by differences in species, cell types, and experimental conditions.

On the other hand, the rapid advancement of epigenetic editing and sequencing technologies is set to revolutionize our understanding of the forces shaping the methylome. By artificially modifying DNAm at key genomic hubs, researchers can uncover changes that are not confined to the local region but resonate across the entire epigenome.^42^ Given the extraordinary complexity of these interactions, innovative bioinformatic tools—including machine learning approaches and AI—will be crucial for decoding the genome-wide interplay. Ultimately, exploring the epigenetic crosstalk could lead to groundbreaking therapeutic strategies aimed at rectifying aberrant epigenetic landscapes implicated in various diseases. We stand at the threshold of unraveling the complex web that forms the epigenetic network—an exciting and transformative frontier in contemporary biology.

## Acknowledgments

I would like to acknowledge Dr. Kira Zeevaert, Dr. Ian O. Shum, Dr. Wouter Hubens, René Krüger, and Prof. Dr. Thomas Stiehl (all RWTH Aachen Medical School) for critical feedback on this manuscript. This research was supported by the 10.13039/501100001659Deutsche Forschungsgemeinschaft (DFG: 363055819/GRK2415, WA 1706-12-2/CRU344/417911533, WA1706-14-1/458369830, WA1706-17-1/561150360, and SFB 1506-1/450627322), the José Carreras Foundation (DJCLS 03 R/2024), and the 10.13039/501100002347Federal Ministry of Education and Research (VIP+: 03VP11580).

## Author contributions

W.W. conceived and wrote this review.

## Declaration of interests

W.W. is cofounder of the company Cygenia GmbH (www.cygenia.com), which provides services for epigenetic analyses to other scientists.
